# Four‐Year Cognitive Outcomes in Multiple Sclerosis: Associations With Lesion Myelin Integrity

**DOI:** 10.1002/acn3.70577

**Published:** 2026-11-04

**Authors:** Iliana Pliska‐Bloch, Hannah Schwartz, Kimberly H. Markowitz, Abhishek Jaywant, Christopher D. Hildenbrand, Jai Perumal, Ulrike W. Kaunzner, Thanh D. Nguyen, Sandra Hurtado Rúa, Susan A. Gauthier

**Affiliations:** ^1^ Department of Neurology Weill Cornell Medicine New York New York USA; ^2^ Department of Psychiatry Weill Cornell Medicine New York New York USA; ^3^ Mathematics and Statistics Cleveland State University Cleveland Ohio USA; ^4^ Department of Radiology Weill Cornell Medicine New York New York USA; ^5^ Feil Family Brain and Mind Institute Weill Cornell Medicine New York New York USA

**Keywords:** cognition, imaging, multiple sclerosis, myelin water fraction

## Abstract

**Objectives:**

Cognitive impairment is prevalent in patients with multiple sclerosis (MS) but whether lesion myelin integrity influences future cognitive outcomes remains unclear. We investigated the relationship between baseline lesion myelin water fraction (MWF) and cognitive performance 4 years later.

**Methods:**

We conducted a longitudinal study of 118 patients with MS. Cognitive function was evaluated annually for 5 years using the symbol digit modalities test (SDMT), California verbal learning test–II (CVLT‐II) immediate recall, and brief visuospatial memory test–revised (BVMT‐R). Lesion level MWF maps were the primary variable of interest, while paramagnetic rim lesions (PRLs) and structural imaging measures were included as covariates. We used a functional linear regression model with both scalar and functional covariates, followed by a stepwise variable selection procedure.

**Results:**

After adjustment for baseline cognition and clinical covariates, participants with higher baseline lesion‐level MWF values in the mid‐range of the lesion distribution exhibited better SDMT at 4 years (*p* = 0.006). Greater heterogeneity in lesion‐level MWF was associated with worse visuospatial memory performance on the BVMT‐R at Year 4. Conversely, lesion MWF was not associated with CVLT‐II performance. Additional covariates in the final models included PRLs and cortical thinning for SDMT, PRLs and disease duration for BVMT‐R, and thalamic atrophy for CVLT‐II.

**Interpretation:**

Lesion myelin integrity was associated with future cognitive performance, particularly in domains of processing speed and visuospatial memory. These findings suggest that lesion demyelination contributes to cognitive outcomes in MS, which may provide a potential focus for future work improving risk stratification for cognitive impairment.

## 1 Introduction

Multiple sclerosis (MS) is a chronic inflammatory and neurodegenerative disease of the central nervous system characterized by immune‐driven demyelination and axonal damage in the brain and spinal cord. These pathological processes manifest as lesions which can be seen on magnetic resonance imaging (MRI). Cognitive impairment is highly prevalent in patients with MS and is associated with a more aggressive disease course with greater disability progression over time [1]. Cognitive deficits can emerge early, even in the absence of other neurological symptoms, but they typically worsen as the disease advances [2]. Cognitive performance varies widely among individuals with MS; however, the most characteristic deficits involve processing speed, followed by impairments in learning and memory [3]. Although gray matter damage [4, 5, 6, 7] and altered white matter integrity [8] are associated with cognitive decline, the variance accounted for is relatively modest [9, 10]. Lesion‐specific pathological features, like paramagnetic rim lesions (PRLs), may provide additional insight into the mechanisms underlying cognitive dysfunction. PRLs are chronically demyelinating lesions with a dense rim of iron laden macrophages, indicative of continued inflammation. Previous research has also demonstrated an association between PRLs and worse cognition compared to patients without these lesions, with deficits in processing speed and memory [11, 12]. These findings suggest that characteristics of individual lesions, beyond conventional measures of lesion burden, may contribute to cognitive dysfunction in MS.

Focal demyelination is the pathological hallmark of MS; however, its effects extend beyond individual lesions, as chronically demyelinated axons lead to mitochondrial dysfunction, axonal instability and eventual neuronal degeneration [13]. While remyelination can partially restore tissue integrity, its extent varies considerably across lesions and between individuals [14, 15]. This heterogeneity in lesion myelin integrity may be particularly relevant to cognition. Cognitive dysfunction is thought to arise from disruption of distributed neural networks rather than damage to isolated brain regions [16]. Therefore, the cumulative pattern of demyelination and remyelination across lesions may provide important information about the overall integrity of brain networks supporting cognitive function.

Recent advances in quantitative imaging allow for the in vivo assessment of myelin content within MS lesions. Studies using myelin‐sensitive imaging measures have reported associations between lesion myelin integrity and physical disability, including expanded disability status scale (EDSS) scores [17, 18]. However, the relationship between lesion myelin damage and cognitive outcomes remains poorly understood. Moreover, prior studies have generally summarized lesion myelin using a single patient‐level metric, such as the mean or median value across all lesions [19]. While informative, these approaches do not capture the substantial heterogeneity in myelin content that exists across lesions within an individual patient [20, 21].

Myelin water fraction (MWF) imaging provides an opportunity to explore the impact of lesion demyelination on clinical outcomes [15, 22]. Although studies have demonstrated altered MWF along white matter tracts and its association with cognitive performance [23], evidence directly linking lesion‐specific myelin measurements, using any imaging modality, to cognition is lacking. Furthermore, because demyelination and remyelination vary substantially across lesions, patient‐level summary measures may not fully capture lesion pathology. Characterizing the distribution of lesion myelin integrity across lesions may provide a more sensitive marker of the cumulative effects of tissue injury and repair [22, 23]. In this study, we investigated whether baseline lesion‐level MWF distributions were associated with cognitive performance 4 years later in patients with MS. We hypothesized that greater lesion demyelination and greater heterogeneity in lesion myelin integrity would be associated with worse future cognitive outcomes.

## 2 Subjects and Methods

### 2.1 Participants

This observational study was conducted in a cohort of individuals with MS who underwent MRI and comprehensive cognitive evaluations at baseline and annually for 4 years. Subjects were recruited from 2018 to 2019 from the Weill Cornell Medicine Judith Jaffe MS Center. Follow‐up occurred annually with final study visits completed between September 2022 and May 2024.

This study initially enrolled 177 participants with MS who met the following inclusion criteria: (1) 2017 McDonald criteria, (2) age ≥ 18 years, (3) participation in our research repository with willingness to undergo annual cognitive evaluation concurrent with MRI, and (4) no concurrent medical conditions which could affect their cognitive performance. The patients enrolled had a diagnosis of clinically isolated syndrome (CIS), relapsing–remitting MS (RRMS), or secondary progressive MS (SPMS). Of these, 118 participants completed both baseline and four‐year follow‐up assessments and were included in the present analysis (CIS *n* = 6 [5.1%], RRMS *n* = 105 [89%], SPMS *n* = 7 [5.9%]). The remaining participants were lost to follow‐up, largely due to the COVID‐19 pandemic. Therefore, this analysis focused on the remaining sample of 118 patients. Cognitive function was evaluated utilizing The *Brief International Cognitive Assessment for Multiple Sclerosis (BICAMS)*, which is composed of three assessments: oral symbol digit modalities test (SDMT), California verbal learning test‐II (CVLT‐II) immediate recall (Total of Trials 1–5), and brief visuospatial memory test‐revised (BVMT‐R) immediate recall (Total of Trials 1–3) [24]. BICAMS is a reliable, valid, and frequently used cognitive assessment in MS populations. SDMT assesses processing speed, CVLT‐II measures verbal learning and short‐term memory, and BVMT‐R examines visuospatial learning and short‐term memory. A lower score for each cognitive assessment is associated with a lower cognitive performance. The same version of the battery was performed each year. The following clinical data were also collected for all patients: age, sex, disease duration from initial symptoms, duration on current disease‐modifying treatments (DMT), expanded disability status scale (EDSS) score, and disease subtype. Treatments were groups as the following: (a) high efficacy: ocrelizumab, alemtuzumab, natalizumab, rituximab and fingolimod; (b) low efficacy: dimethyl fumarate, teriflunomide, glatiramer acetate, beta‐interferon, and monthly intravenous immunoglobulin; and (c) none.

The study was approved by an ethical standards committee on human experimentation at Weill Cornell Medicine, and written informed consent, which provides permission to store and analyze clinical and MRI data, was obtained from all patients according to the Declaration of Helsinki.

### 2.2 MRI Scanning Protocol

Imaging was performed on clinical whole‐body 3 T Magnetom scanners (Siemens Medical Solutions USA, Malvern, PA) using a product 20‐channel head/neck coil. The MRI protocol consisted of 3D T1‐weighted (T1w) sequence for anatomical structure, 2D T2‐weighted (T2w) turbo spin echo and 3D T2w fat‐saturated fluid attenuated inversion recovery (FLAIR) sampling perfection with application optimized contrasts using different flip‐angle evolutions (SPACE) sequences for lesion detection, gadolinium‐enhanced 3D T1w sequence, 3D multi‐echo gradient echo (GRE) sequence for quantitative susceptibility mapping (QSM) and a 3D fast acquisition with spiral trajectory and adiabatic T2prep (FAST‐T2) [25] for MWF mapping. The detailed acquisition parameters were as follows: (1) sagittal 3D T1w MPRAGE: Repetition Time (TR)/Echo Time (TE)/Inversion Time (TI) = 2300/2.3/900 ms, flip angle (FA) = 8°, GRAPPA parallel imaging factor (R) = 2, voxel size = 1.0 × 1.0 × 1.0 mm^3^; (2) axial 2D T2w turbo spin echo: TR/TE = 5840/93 ms, excitation FA = 90°, refocusing FA = 140°, turbo factor = 18, *R* = 2, number of signal averages (NSA) = 2, voxel size = 0.5 × 0.5 × 3 mm^3^; (3) sagittal 3D T2w FLAIR SPACE: TR/TE/TI = 7600/448/2450 ms, excitation FA = 90°, variable refocusing flip angle = yes, turbo factor = 273, *R* = 4, voxel size = 1.0 × 1.0 × 1.0 mm^3^; (4) axial 3D multi‐echo GRE: TR/TE1/ΔTE = 48.0/6.3/4.1 ms, number of TEs = 10, FA = 15°, *R* = 2, voxel size = 0.75 × 0.75 × 3 mm^3^; and (5) axial 3D FAST‐T2: spiral TR/TE = 7.8/0.5 ms, nominal T2prep times = 0 (T2‐prep turned off), 7.5, 17.5, 67.5, 147.5, and 307.5 ms, T1 saturation recovery time (wait time between the saturation pulse at the end of spiral readout and the next T2prep) = 2 s, FA = 10°, readout bandwidth = 1042 Hz/pixel, number of spiral leaves per stack = 32, number of spiral leaves collected per T2prep = 64, voxel size = 1.3 × 1.3 × 5 mm^3^.

### 2.3 QSM and MWF Map Extraction

QSM was reconstructed from complex GRE images using the fully automated MEDI toolbox (available at https://pre.weill.cornell.edu/mri/pages/qsm.html) [26] and an automatic uniform cerebrospinal fluid zero reference (MEDI+0) algorithm [27]. Whole‐brain MWF maps were computed from the Gibbs‐ and noise‐corrected FAST‐T2 image data using a multi‐voxel three‐pool nonlinear least‐squares fitting algorithm with a Laplacian local spatial smoothness constraint [25]. The relative MWF was calculated as the ratio of the signal of the myelin water to the total water signal within a voxel.

### 2.4 Image co‐Registration

For each time point, T1w image was brain‐extracted, intensity normalized and aligned to the FreeSurfer T1w conformed space (256 × 256 × 256 1 mm isotropic voxels) using FreeSurfer v6 [28]. At each time point, images were registered cross‐sectionally to the subject‐specific FreeSurfer T1 image using ANTs [29] with a rigid‐body transformation model (translation and rotation) and mutual information similarity metric. For longitudinal registration, the subject‐specific FreeSurfer T1 image at each time point was aligned to the baseline subject‐specific FreeSurfer T1 image using rigid‐body transformation with mutual information similarity metric, followed by non‐linear deformation using ANTs [29] with cross‐correlation similarity metric. The resulting transformation matrix and warp field were concatenated with the cross‐sectional transformation to bring each image to the baseline FreeSurfer T1 space. Spline interpolation was used in all registrations. This automated registration approach was applied consistently to all images, and the registration outputs were visually inspected in ITK‐SNAP for quality control.

### 2.5 Brain Tissue Segmentation

FreeSurfer [30] was utilized for the segmentation of white matter (WM) and gray matter (GM) on T1w images. The GM segmentation masks were checked, and lesions were manually filled in where necessary to prevent misclassification due to T1‐hypointensities associated with lesions [31]. Cortical thickness was computed using FreeSurfer, and thalamic volumes for all subjects were obtained using the fully automated surface reconstruction and volumetric segmentation pipeline. This automated approach has been described in detail previously [32]. Longitudinal changes were derived using the FreeSurfer longitudinal processing pipeline. Since structures may scale with the general head size, the thalamic volume was normalized to the overall intracranial volume (estimated total intracranial volume (eTIV) in FreeSurfer).

### 2.6 Lesion Segmentation and Paramagnetic Rim Lesion Identification

MS lesions were automatically identified and segmented on the FLAIR image [33], which was followed by manual editing and creation of individual lesion labels. T1w and T2w images were referenced, as needed, during the editing process to define lesion boundaries, especially in the case of confluent lesions. Lesion masks were manually edited by trained research staff and reviewed by the senior author (SG) to confirm accuracy. PRLs were identified on QSM [34, 35] by a consensus of two independent reviewers, and a third independent reviewer resolved any discrepant lesions. These reviewers consisted of experts with multiple years of experience in the identification of PRLs. Lesions with partial or complete rims were considered PRLs, in accordance with the most recent consensus statement on imaging chronic active lesions [36].

### 2.7 Statistical Analysis

The primary endpoint was SDMT score at 4‐year follow‐up, modeled as a function of scalar and functional covariates (Figure 1) [37, 38]. Scalar covariates included baseline SDMT, age, disease duration, sex, and treatment as adjusting covariates, as well as baseline and follow‐up MRI covariates including change in cortical thickness (Δct = cortical thickness at year 4 minus cortical thickness at baseline), change in normalized thalamic volume (Δntv = normalized thalamic volume at year 4 minus normalized thalamic volume at baseline), presence of PRLs at baseline, and lesion burden measured by the number of FLAIR‐hyperintense lesions and total FLAIR lesion volume. Because patients with MS differ considerably in lesion number and lesion‐specific MWF values, summarizing lesion myelin integrity with a single value may obscure important information. We therefore modeled lesion MWF as a functional covariate, which preserves the full distribution of lesion MWF values within each participant and captures variability across lesions that is not reflected in summary statistics alone. This same framework was also applied to evaluate secondary cognitive outcomes, including CVLT‐II and BVMT‐R scores at follow‐up.

**FIGURE 1:**
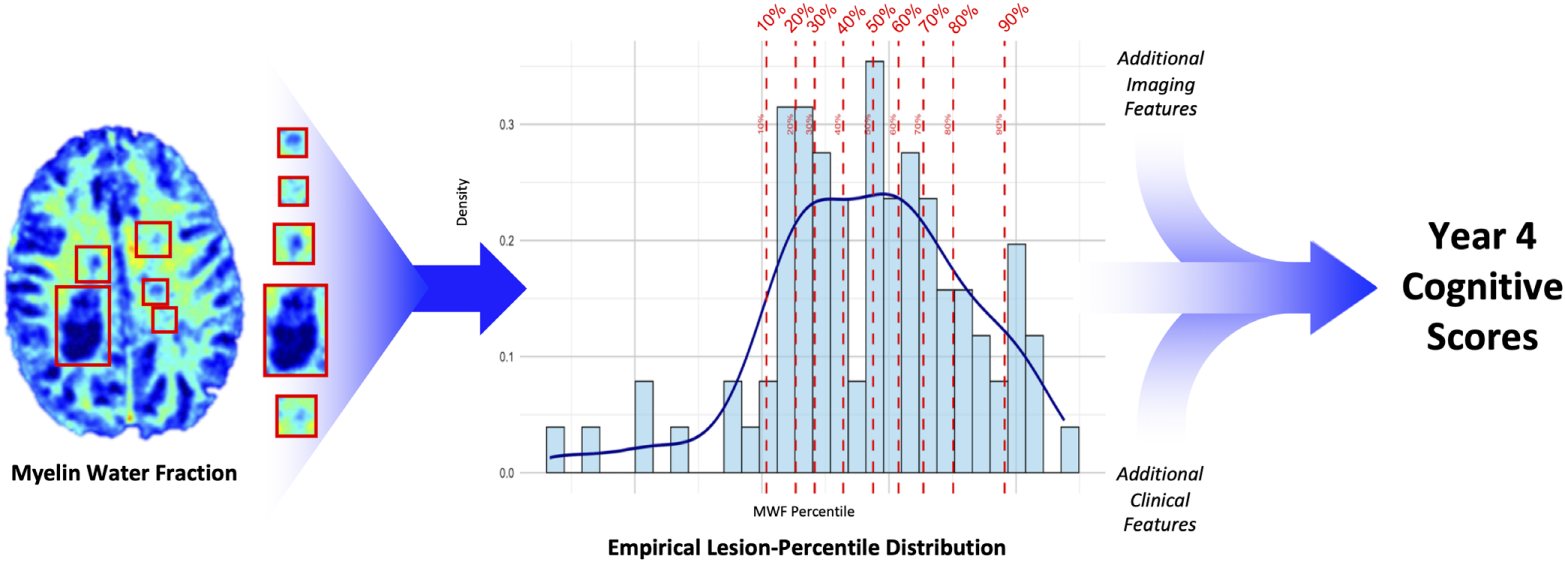
Graphical representation of scalar and functional variables included in statistical modeling. This figure demonstrates how the functional and scalar covariates were included in the model. Using baseline MWF imaging and lesion masks, MWF lesion percentages were summarized using percentiles, creating an empirical density of MWF for each patient. Additional imaging variables included total lesion volume, number of lesions, cortical thickness, normalized thalamic volume, and the presence of PRLs. Clinical and demographic features included age, sex, disability represented by EDSS, disease duration, disease‐modifying therapy, and baseline cognitive scores. All variables above were included in the modeling strategy. The final model analyzing their association with cognitive scores at year 4 included only significant variables. EDSS, expanded status disability scale; MWF, myelin water fraction; PRLs, paramagnetic rim lesions.

We modeled the functional covariate by projecting the lesion MWF percentile function onto B‐spline and Bernstein basis using the R package *fda*. These bases were chosen for their suitability for modeling functions on percentile intervals, providing a balance between flexibility and numerical stability. Candidate models varying in basis type and number of basis functions were compared using adjusted R^2^, AIC, BIC, GCV, condition number, and inspection for aliased coefficients. Top‐performing models were further refined using stepwise regression with stepAIC from the MASS package in R. The final model retained statistically significant variables. Multicollinearity was assessed, and model assumptions were checked to evaluate robustness.

The functional regression framework is described in full in the Supporting Information. Briefly, each patient's lesion MWF distribution was represented as an empirical percentile function and entered as a functional covariate, with the coefficient function β(q) expanded on a B‐spline basis (Section S1). The choice of basis family and dimension is detailed in Section S2. Robustness of the findings was assessed through a series of sensitivity analyses (Section S3), including robustness to modeling framework (Sections 3.1, 3.2). Sensitivity to scalar variable selection is included in (S3.4), and model diagnostics in (S3.5).

We use a significant level of 0.05. All analysis was performed using R: A language and environment for statistical computing.

## 3 Results

### 3.1 Patient Cohort Over Four Years

The 118 patients at baseline had a mean age of 42.84 ± 9.78 years, median EDSS 1.00 [IQR 0.00–2.00], and mean disease duration of 11.15 ± 7.39 years. Forty‐five patients (38.1%) had at least one PRL at baseline. Baseline characteristics are presented in Table 1. Average follow up time was 50 months (SD = 4.12), with the variability largely being explained by the impact of the COVID‐19 pandemic on patients' annual appointments. Across the 4‐year follow‐up, BVMT‐R remained stable (*p* = 0.226), whereas SDMT and CVLT‐II demonstrated a modest mean increase of 2.24 points (*p* < 0.01) and 3.81 points (*p* < 0.01), respectively. Baseline and follow‐up cognitive scores and range, as well as EDSS scores and range are presented in Table S10 as supporting material. Over 4 years, there were 67 new FLAIR lesions observed across 24 patients, and 4 new PRLs observed across 4 patients. The majority of these patients (69.44%) were on either low‐efficacy DMTs or no DMT. Both cortical thickness (*p* < 0.01) and thalamic volume (*p* < 0.01) on average decreased over time.

**TABLE 1:** Description of cohort at baseline.

Demographic and clinical characteristics at baseline	*N* = 118
Age, mean (SD)	42.84 (9.78)
Sex	
Male, *n* (%)	35 (29.7)
Female, *n* (%)	83 (70.3)
Diagnosis	
CIS, *n* (%)	6 (5.1)
RRMS, *n* (%)	105 (89)
SPMS, *n* (%)	7 (5.9)
Disease‐modifying treatment	
High efficacy, *n* (%)	63 (53.38)
Low efficacy, *n* (%)	45 (38.13)
None, *n* (%)	10 (8.47)
Disease duration, mean (SD)	11.15 (7.39)
EDSS, median [IQR]	1 [0, 2.00]
Imaging characteristics	
FLAIR total lesion volume (mm^3^), mean (SD)	5715.05 (6738.80)
No. of FLAIR lesions, mean (SD)	28.74 (23.07)
No. of patients with 31 PRL, *n* (%)	45 (38.1)
No. of paramagnetic rim lesions, median [IQR]	0 [0, 1]
Cortical thickness (mm), mean (SD)	2.43 (0.09)
Normalized thalamic volume, mean (SD)	0.01 (0.00)
Cognitive scores	
SDMT, mean (SD)	56.11 (11.52)
BVMT‐R, mean (SD)	24.16 (6.03)
CVLT‐II, mean (SD)	52.72 (8.50)

Abbreviations: BVMT‐*R*, brief visuospatial memory test–revised; CIS, clinically isolated syndrome; CVLT‐II, California verbal learning test—second edition; EDSS, expanded disability status scale; PRL, paramagnetic rim lesion; RRMS, relapsing–remitting multiple sclerosis; SDMT, symbol digit modalities test; SPMS, secondary progressive multiple sclerosis.

### 3.2 Lesion MWF Associated With Future Cognitive Performance

We identified several brain features associated with SDMT performance at 4 years after adjusting for baseline cognitive scores and patient‐level characteristics. In the functional regression model, baseline SDMT (*β* = 0.6730, *p* < 0.001) and age (*β* = −0.2940, *p* < 0.001) were statistically significant adjusting covariates. After accounting for these factors, SDMT at 4 years was significantly associated with the presence of at least one PRL at baseline (*β* = −0.3973, *p* = 0.0187), change in cortical thickness (*β* = 43.8824, *p* = 0.0139), and one coefficient related to the lesion‐level MWF distribution at baseline (θ = 4.7175, *p* = 0.0058; Figure 2A). This coefficient reflects a positive association between lesion myelin content within the mid‐range of the MWF distribution and SDMT performance. Figure 2A illustrates the MWF basis coefficients that were statistically significant, indicating the portions of the lesion myelin distribution most strongly associated with SDMT outcomes. These coefficients were subsequently transformed to facilitate interpretation (Figure 2B). The effect was localized to a mid‐range of lesion MWF values (9.00%–10.34%; Figure 2C). Within this range, *β*(q) reached its maximum (approximately 2), indicating that higher lesion MWF in the mid‐range was positively associated with SDMT. The average pointwise contribution to SDMT (Figure 2D) shows a strong association in the mid‐range and a near zero or negative association at the extremes. Figure 3 presents representative examples of patients with differing baseline lesion MWF distributions and corresponding SDMT outcomes at 4 years. Overall, higher lesion MWF within the mid‐range of the baseline distribution was associated with better SDMT performance at 4 years, after considering other imaging and clinical covariates. The marginal effects plot (Figure 4) shows model estimates of SDMT at 4 years as a function of mid‐range lesion MWF, at representative levels of baseline cognition; predicted SDMT increased with higher mid‐range MWF across all baseline strata, consistent in direction and magnitude.

**FIGURE 2:**
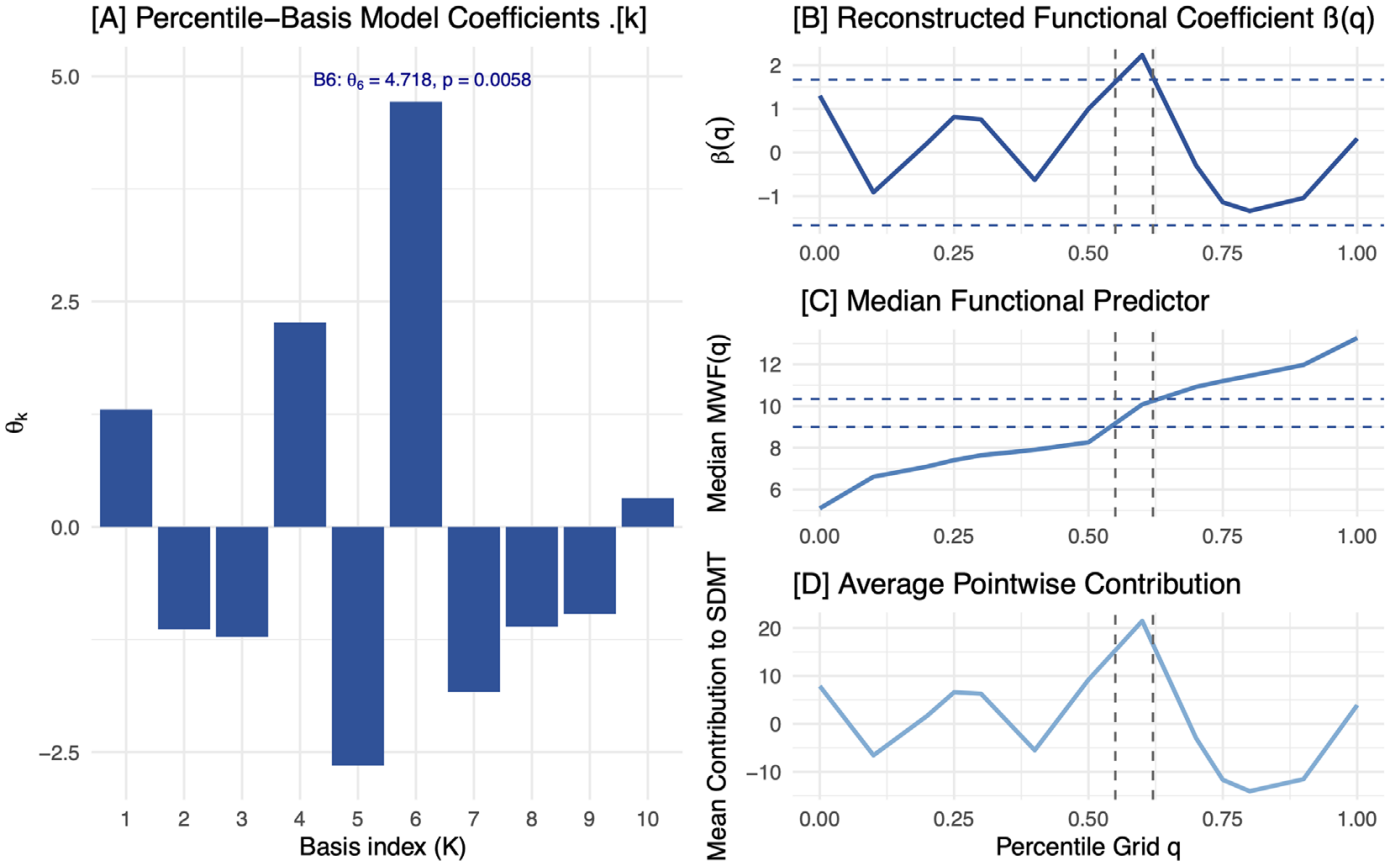
Percentile‐basis functional effects of MWF on SDMT. The 4 panels depict how the functional coefficient *β*(*q*) and the covariate lesion MWF(q) jointly determine contribution values across the percentile grid, with θ[k] representing the corresponding basis‐space model coefficients. Contribution peaks arise where *β*(*q*) reaches high values. Dashed vertical and horizontal lines indicate percentile boundaries. [A] Percentile‐basis model coefficients (θ[k]) plot is the plot of estimated coefficients for each percentile basis function. Positive values indicate a positive effect of the corresponding basis on SDMT; negative values indicate a negative effect. The annotation shows the only significant coefficient, $\;\theta_{6},$, along with its *p*‐value. [B] Reconstructed functional coefficient *β*(*q*) plot shows the effect of the functional covariate, MWF at each percentile of the lesion distribution. Dashed vertical lines indicate the boundaries of the significant quantile regions. [C] The median quantile function across patients, representing a typical ‘patient lesion MWF distribution in this cohort. The vertical dashed lines mark the percentile range over which the coefficient function is maximal, and the horizontal dashed lines mark the corresponding MWF values (9.00%–10.34%), which are read from the vertical axis. [D] Average pointwise contribution is the pointwise product of *β*(*q*) and MWF(q), showing where MWF most strongly drives SDMT. MWF, myelin water fraction; SDMT, symbol digit modalities test.

**FIGURE 3:**
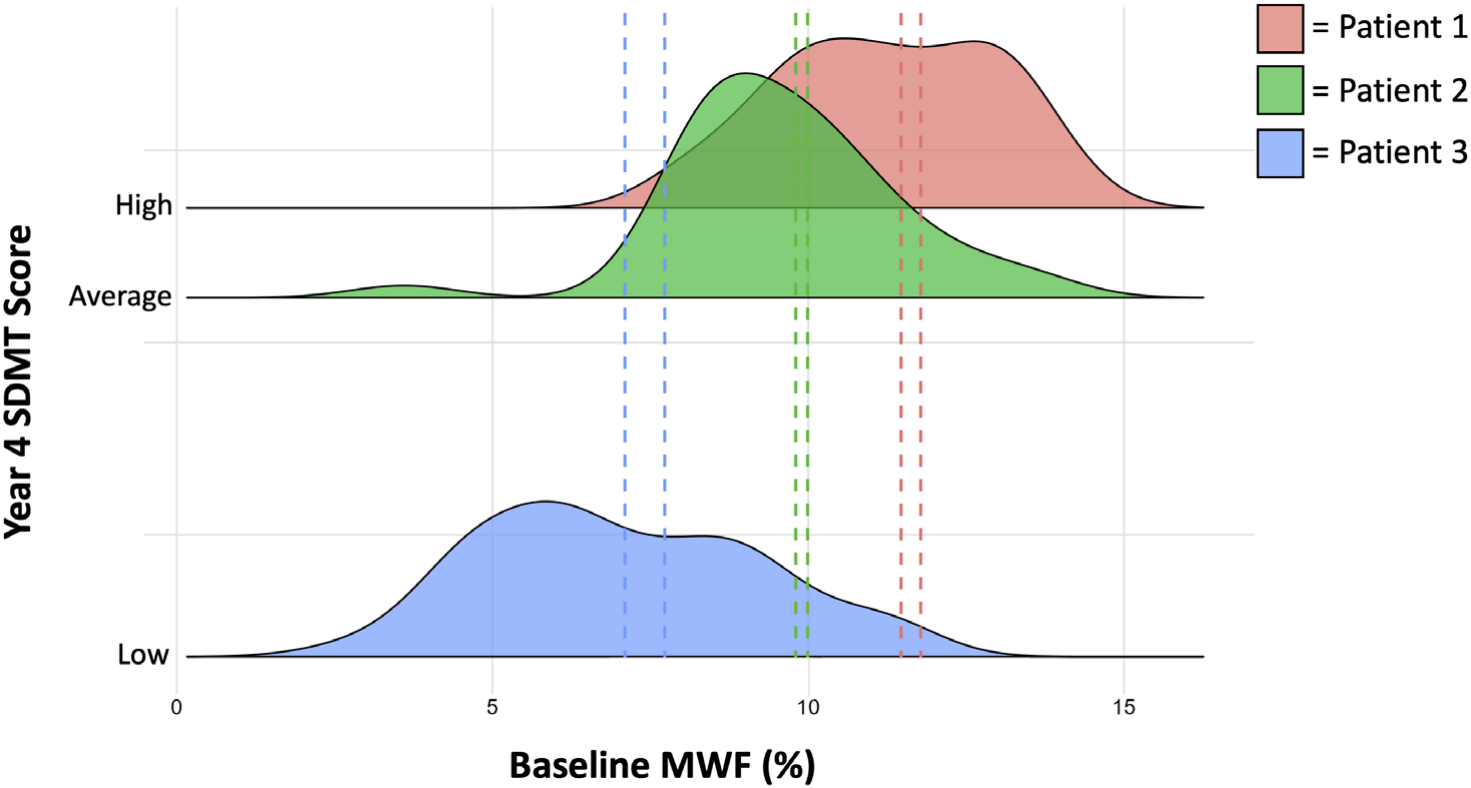
Patient examples of lesion MWF and SDMT at 4 years. This figure presents the raw distributions of lesion MWF percentage from three representative patients. The patients were selected to represent a low SDMT score, a medium SDMT score, and a high SDMT score. Each individual patient has different baseline lesion MWF values within the mid‐range, which is highlighted by the dotted lines. These examples illustrate how higher MWF values in the mid‐range of the distribution are associated with higher SDMT performance at 4 years. MWF, myelin water fraction; SDMT, symbol digit modalities test.

**FIGURE 4:**
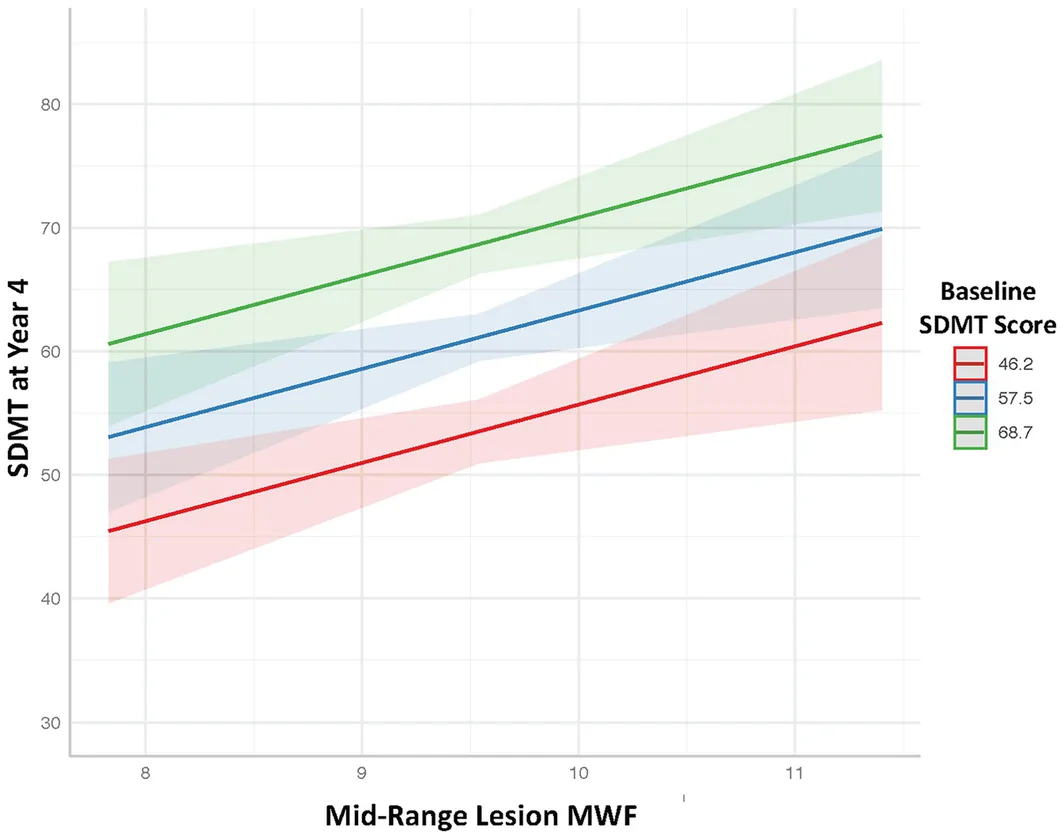
Expected SDMT scores at 4 years. Expected SDMT scores at year 4 as a function of lesion MWF % stratified by baseline SDMT score percentile. These marginal effects plots illustrate the relationship between MWF (mid‐range, functional component: B6) and SDMT quartiles at year 4, stratified by baseline SDMT scores (red = 46.2, Q1; blue = 57.5, Q2; and green = 68.7, Q3). Each color represents a different baseline SDMT level. The figure illustrates the marginal effect of baseline SDMT and MWF on SDMT outcomes. MWF, myelin water fraction; PRLs, paramagnetic rim lesions; SDMT, symbol digit modalities test.

In our secondary analysis, after adjusting for baseline BVMT‐R (*β* = 0.8100, *p* < 0.001) and disease duration (*β* = −0.1726, *p* = 0.0050), BVMT‐R score at 4 years was associated with the presence of at least one PRL at baseline (*β* = −2.9178, *p* = 0.0035). Several functional MWF coefficients (θ_2_, θ_3_, θ_8_, and θ_10_) reached statistical significance at the 0.10 level (Figure 5A) and corresponded to the tails of the lesion myelin distribution, contributing to BVMT‐R performance.

**FIGURE 5:**
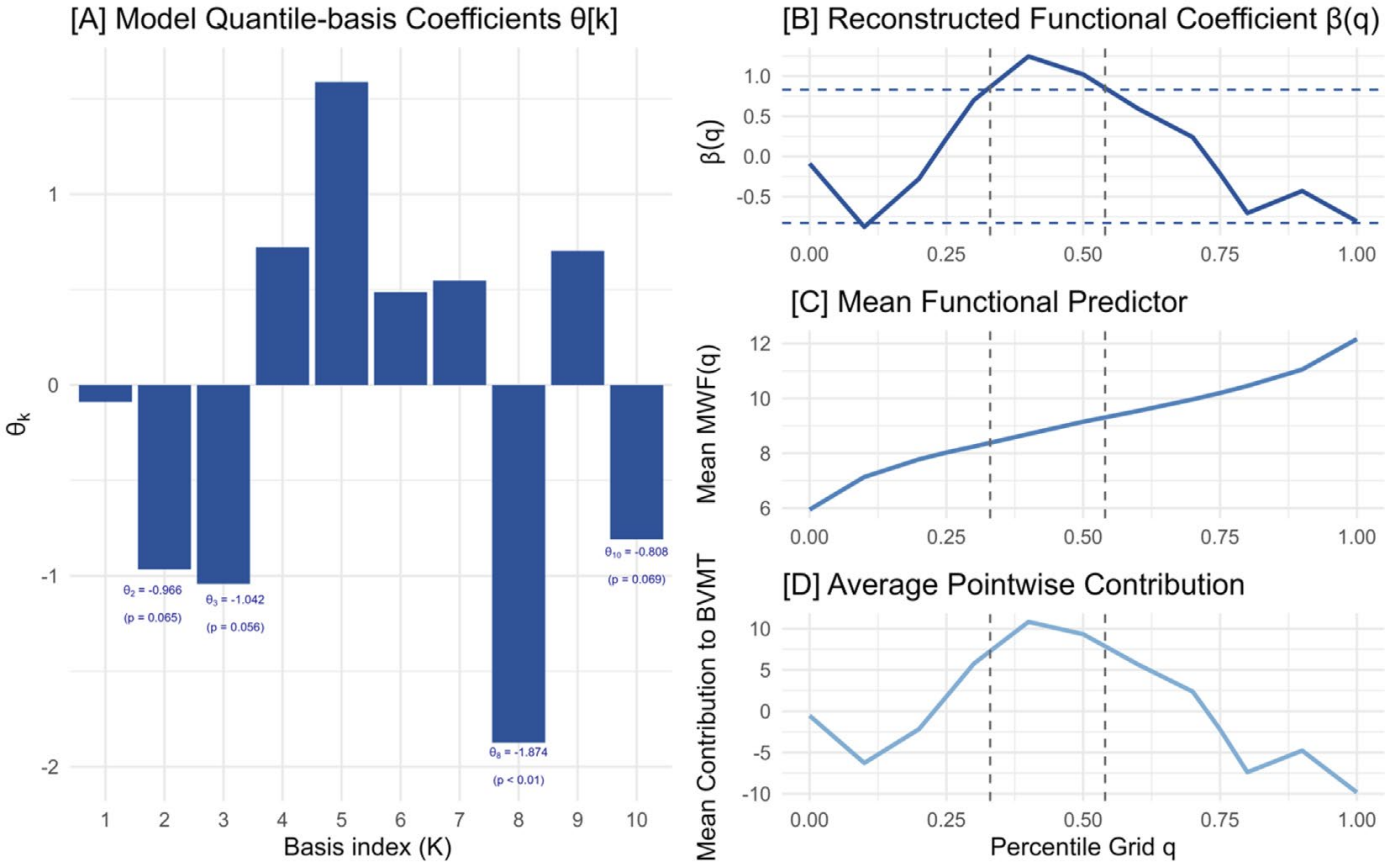
Percentile‐basis functional effects of MWF on BVMT‐R. The 4 panels depict how the functional coefficient *β*(*q*) and the covariate lesion MWF(q) jointly determine contribution values across the percentile grid, with θ[k] representing the corresponding basis‐space model coefficients. Contribution peaks arise where *β*(*q*) is at extreme values. Dashed vertical and horizontal lines indicate percentile boundaries. [A] Percentile‐basis model coefficients (θ[k]) plot is the plot of estimated coefficients for each percentile basis function. Positive values indicate a positive effect of the corresponding basis on BVMT‐R; negative values indicate a negative effect. The annotation shows the only significant coefficients ($\theta_{2}$, $\theta_{3},\theta_{8},\text{and}\;\theta_{10}$), along with its *p*‐values. [B] Reconstructed functional coefficient *β*(*q*) plot shows the effect of the functional covariate, MWF, at each percentile of the lesion distribution. Dashed vertical lines indicate the significant quantile regions. [C] Mean functional covariate MWF(q) is the average value of the percentile MWF across all subjects. [D] Average pointwise contribution is the pointwise product of *β*(*q*) and MWF(q), showing where MWF most strongly drives BVMT‐R. BVMT‐*R*, brief visuospatial memory test—revised; MWF, myelin water fraction.

This pattern indicates that greater heterogeneity in lesion MWF values was associated with lower BVMT‐R scores at follow‐up, with an estimated point‐wise reduction of up to 9.82 points (Figure 5D). Figure 5B–5D show that within the lower mid‐range (25th–50th percentiles) of lesion MWF values (8.07%–9.47%), MWF was associated with a higher BVMT‐R score at follow‐up, conditional on other covariates, corresponding to a cumulative marginal contribution of up to 10.83 points.

CVLT‐II at 4 years was adjusted by baseline CVLT‐II (*β* = 0.8453, *p* < 0.001) and associated with change in normalized thalamic volume (*β* = 3831.82, *p* = 0.0439). No MWF functional coefficients were significant at the 0.10 level for CVLT‐II. These results demonstrate that lower baseline CVLT‐II and greater reductions in normalized thalamic volume were associated with lower verbal memory performance at follow‐up.

## 4 Discussion

Cognitive impairment in MS has been an unpredictable and poorly understood aspect of the disease with significant functional consequences and without adequate treatment options. In this study, we found that positive cognitive outcomes at 4 years were associated with higher ranges of lesion‐level myelin damage after controlling for other imaging covariates known to associate with cognitive impairment. These findings suggest that evaluating cognition in MS requires attention to both widespread neurodegenerative changes and lesion‐based assessment. While global atrophy, specifically thalamic volume loss, has traditionally been emphasized, our results show that a lesion‐level analysis may also contribute to the understanding of long‐term cognitive vulnerability.

Among the cognitive measures in this study, the SDMT demonstrated the strongest and most consistent associations with MRI features. This aligns with prior literature showing that the SDMT is one of the most sensitive and reliable markers of MS‐related cognitive decline [39]. Our models accounted for baseline cognitive performance, whereas approaches that do not may reflect between‐subject differences in baseline cognition rather than associations with subsequent cognitive performance. The persistence of the association between SDMT and MRI features after baseline adjustment suggests that imaging markers capture clinically meaningful variation in cognition, underscoring their relevance for understanding this disease‐related impairment. While the SDMT is typically used as a measure of processing speed, multiple other cognitive skills, including working memory, attention, and language access, are required to complete the test [40]. For this reason, cognitive impairments across multiple domains may be detected through the SDMT, which could explain its strong fit based on the model R^2^ values (see Table S8). Interestingly, recent work has shown that even when their cognitive speed is normal, patients still scored below average on the SDMT [41]. More research is needed to better understand how cognitive impairment initially presents in MS, but this study helps close this gap by demonstrating a clear association between cognition and lesion MWF. To better apply these results clinically, additional research is needed to determine whether remyelination treatment strategies support cognitive function.

### 4.1 Lesion MWF and Cognitive Outcomes

In this study, greater preservation of mid‐range lesion MWF at baseline was associated with better cognitive performance after 4 years of follow‐up. MWF is an indirect measure of myelin content but offers higher specificity than other clinically feasible quantitative MRI metrics [42]. Prior studies have shown that increased MWF heterogeneity along major white matter tracts, consistent with greater tissue damage, is associated with cognitive impairment [23]. To our knowledge, no prior studies have examined the extent of myelin damage within individual lesions using MWF and its relationship to cognitive performance [43]. Previous research has demonstrated that lesions disrupting structural connectivity within specific white matter tracts are associated with poorer performance on the SDMT [44]. It has also been shown that cognition may be influenced by MWF changes in normal appearing white matter. Our findings extend this literature by focusing on the extent of myelin damage within lesions themselves.

We assessed lesion MWF as a functional covariate at the voxel level and summarized it using the empirical lesion‐percentile distribution. While complex, this approach moves beyond global lesion averages and captures heterogeneity in lesion pathology, allowing a more detailed characterization of how lesion‐specific myelin damage relates to cognitive outcomes. Although the functional component corresponding to mid‐range MWF values was significant for SDMT and may be less intuitive clinically, this range reflects lesion values consistent with relatively preserved myelin. This analysis emphasizes the impact of myelin loss even when lesion myelin remains relatively preserved, as those with lower myelin values in the mid‐range were associated with worse performance on the SDMT at year 4. The association between greater heterogeneity in lesion MWF and worse BVMT‐R performance is less straightforward but may indicate variability in myelin integrity across lesions. Had we used mean MWF across all a patient's lesions, we may not have been able to see this association. While its clinical interpretation will benefit from further analysis, including the location of lesions, this association supports the analysis of individual lesion MWF in the investigation of what drives cognitive impairment in MS.

Further, our modeling approach offers several advantages: it identifies which lesion MWF percentiles are most strongly associated with cognition, captures localized non‐linear effects that are obscured by global summaries, reduces sensitivity to outliers, and provides a clearer link between lesion pathology and cognitive performance. While further research is still needed to understand the pathological mechanisms driving cognitive impairment, this method goes beyond the existing literature to enhance our understanding of the relationship between lesion‐level pathology and cognitive outcomes, highlighting subtle patterns that may inform targeted interventions. Compared with a global MWF measure, our modeling approach retains the distributional structure of lesion MWF and may better capture localized, complex relationships between lesion pathology and cognitive performance.

PRLs have emerged not only as a strong diagnostic tool, but also as a potential prognostic indicator, with a higher number of PRLs associated with more severe disease progression [45]. PRLs have also been linked to impaired cognitive function in the earliest stages of the disease [11, 46].

Post‐mortem have demonstrated that PRLs exhibit greater myelin damage and evidence of ongoing demyelination at the lesion edge, characterized by an iron‐laden inflammatory rim [47]. These findings suggest that the presence of PRLs may reflect a greater burden of myelin injury and ongoing demyelination, which in turn may relate to poorer clinical outcomes. However, PRLs capture only one aspect of lesion‐level pathology, and more granular measures of myelin integrity may further elucidate how lesion heterogeneity contributes to cognitive outcomes.

### 4.2 Global Brain Features and Cognitive Outcomes

Although cortical gray matter damage [7, 48] has been associated with cognitive decline, the variance accounted for is relatively modest [9, 10]. Among our models, the contribution of cortical thickness was inconsistent and reached significance only for SDMT when accounting for other relevant covariates. Prior studies have reported associations between reduced thalamic volume and poorer cognitive performance, [49, 50]; however, in our analyses, thalamic volume was significantly associated only with CVLT‐II performance and showed a trend‐level association (*p* = 0.062) with SDMT. Furthermore, the current models only found longitudinal assessments of structural measures (e.g., cortical thickness and thalamic volume) to be significant, as opposed to their baseline values. This suggests that progressive tissue loss within gray matter structures, rather than pre‐existing volume differences, may be a more sensitive marker of future cognitive decline. From a clinical perspective, this highlights the importance of dynamic monitoring of neurodegeneration rather than relying solely on cross‐sectional metrics. Patients with ongoing cortical or thalamic loss may represent a subgroup at heightened risk for cognitive deterioration, reinforcing the potential utility of quantitative MRI measures in MS follow‐up and treatment planning.

### 4.3 Limitations

Firstly, this study presents a cohort with relatively mild disease, which may limit the generalizability of these results across patients with longer disease duration or higher EDSS scores. Across the cohort, cognitive performance on both SDMT and CVLT‐II demonstrated a slight increase over the 4 years. This may in part reflect the use of highly effective DMTs in more than half of the participants, and heterogeneity in treatment exposure may also have influenced both imaging and cognitive outcomes. This is notable given prior evidence that highly effective therapies can influence lesion MWF; [51] however, treatment was not significantly associated with any outcomes in our analysis. In addition, education level was not included as a covariate due to incomplete data across the cohort, which may represent a limitation given the potential influence of education on cognitive performance. However, 70% of the cohort had a college education or higher, suggesting that the potential impact of education level on our findings may be limited. Interestingly, there was minimal change in cognitive scores at the group level over 4 years. Practice effects cannot be excluded, as the same version of each cognitive test was administered annually, potentially limiting our ability to detect true changes in cognitive performance. However, 36 of 118 patients (31%) demonstrated a reliable change of ≥ 8 points on the SDMT [52], with 14% showing a decrease and 39% showing an increase, suggesting that meaningful individual‐level changes in cognitive performance were detected despite the potential for practice effects.

Although we employed a lesion‐based analytic framework, lesion location was not incorporated. This may be particularly relevant for MWF measurements, as myelin content varies across cerebral structures. Future studies should focus on developing a normalized MWF atlas and quantifying lesion‐specific deviations relative to age‐matched healthy controls to improve interpretation of regional myelin damage [53]. Average lesion MWF did not show significant change over time. This may reflect limitations in FAST‐T2 spatial resolution and, more broadly, the constraints of global ummary measures. Future work should therefore focus on characterizing myelin changes at the level of individual lesions and evaluating their longitudinal relationship with cognitive function.

Finally, clinical feasibility remains a challenge for larger‐scale translation of these imaging metrics. Standardized acquisition protocols and image‐processing pipelines are required, but these may be difficult outside academic centers. As we work toward developing predictive models to enable individualized precision care, addressing these barriers will be essential.

## 5 Conclusion

Cognitive ability has a substantial impact on quality of life in patients with MS, underscoring the importance of examining this outcome. Identifying imaging markers associated with future cognitive performance may support risk stratification in clinical practice in the future. Our findings indicate that lesion‐level myelin integrity is associated with future cognitive outcomes, alongside conventional structural brain measures. The novelty of these results lies in the application of a granular, lesion‐level assessment of myelin damage that captures heterogeneity across individual lesions, rather than relying on a single summary measure across all lesions. As advanced MRI techniques become more widely available, incorporating MWF‐based measures of myelin integrity may enhance clinical evaluation and impact outcome prediction. Validation in larger, multicenter cohorts remains necessary; however, if confirmed, these imaging features could inform individualized strategies aimed at preserving cognitive function over time.

## Author Contributions

Iliana Pliska‐Bloch and Hannah Schwartz were involved in the acquisition and analysis of the data and drafting a significant portion of the manuscript or figures. Kimberly H. Markowitz and Thanh D. Nguyen was involved in acquisition and analysis of the data, and review of the manuscript. Christopher D. Hildenbrand was involved in statistical modeling and review of the manuscript. Abhishek Jaywant, Jai Perumal, and Ulrike W. Kaunzner. were involved in conception and design of the study and review of the manuscript. Sandra Hurtado Rúa was involved in conception and design of the study, analysis and modeling of data, and drafting a significant portion of the manuscript or figures. Susan A. Gauthier has overseen conception and design of the study, acquisition and analysis of data, and drafting a significant portion of the manuscript or figures.

## Funding

This work was supported by NIH/National Institute of Neurological Disorders and Stroke (NINDS), R01NS104283. NIH/National Center for Advancing Translational Sciences (NCATS), UL1 TR 002384.

## Disclosure

All authors have submitted their disclosure forms as part of submission.

## Conflicts of Interest

The authors declare no conflicts of interest.

## Supporting information


**Table S1:** Estimation procedure.
**Table S2:** Sequential steps in the construction of the functional covariate and estimation of the scalar‐on‐function regression model. Δct = cortical thickness at year 4 minus cortical thickness at baseline, Δntv = normalized thalamic volume at year 4 minus normalized thalamic volume at baseline, PRLs = the presence of paramagnetic rim lesions at baseline (Y/N), n.FLAIR = number of FLAIR hyperintense lesions and total vol. FLAIR = total FLAIR lesion volume. MWF, myelin water fraction; IQR, interquartile range; G, number of percentile grid points; K, number of basis functions; κ, condition number of the design matrix; θk, basis coefficients; βq, coefficient function. Candidate specifications (Step 4) each contained the full set of scalar covariates together with the complete basis block; no term was removed at this stage. Backward AIC‐based selection (Step 7) was applied once and to scalar covariates only, with the basis coefficients held fixed and at no stage eligible for removal. Full results for candidate specifications are given in and sensitivity analyses in Table S3.
**Table S2:** Candidate specifications for the coefficient function.
κ, condition number of the design matrix; Aliased, presence of rank‐deficient (aliased) coefficients. Adj. R^2^, adjusted R^2^; AIC, Akaike information criterion; BIC, Bayesian information criterion; GCV. Sign changes count crossings of zero by the reconstructed *β*(*q*), and roughness is its integrated squared second difference; both were introduced in revision for interpretation and were not used in selection.
**Table S3:** Sensitivity analyses procedure.
The table presents several sensitivity steps relative to the primary model. The primary model is the scalar‐on‐function regression described in Section 1: SDMT at 4 years regressed on the lesion myelin water fraction percentile function, represented by a B‐spline basis, together with baseline cognitive score, age, disease duration, sex, treatment, change in cortical thickness, change in normalized thalamic volume, presence of at least one paramagnetic rim lesion, number of FLAIR lesions, and total FLAIR lesion volume. In every section the functional covariate was retained by design and was at no stage eligible for removal. *β*(*q*), coefficient function; REML, restricted maximum likelihood; MWF, myelin water fraction.
**Table S4:** Variance explained by functional principal components.
Proportion of variance in the lesion myelin water fraction percentile functions accounted for by each functional principal component. Components were obtained by eigenvalue decomposition of the covariance operator of the centered percentile functions across the patients in the analytic sample. FPC is the functional principal component.
**Table S5:** Association between functional principal component scores and four‐year SDMT. Coefficients for functional principal component scores in models of symbol digit modalities test score at 4 years, with 95% confidence intervals in parentheses. Each model additionally contains the full set of scalar covariates listed in Section 1. Models with one to four components are reported so that the stability of each estimate across specifications is apparent; no single number of components was selected. Dash (—) indicates the component was not included in that model. The joint test compares each model with the corresponding model omitting FPC scores. Because component scores are scaled by the eigenvalue decomposition of the covariance operator, coefficient magnitudes are not directly comparable between components. FPC is functional principal component; Adj. R2 is adjusted R2.
**Table S6:** Penalized scalar‐on‐function regression.
Roughness‐penalized scalar‐on‐function regression of four‐year SDMT on the lesion MWF percentile function, adjusting for the full scalar covariate set. The coefficient function was represented with a B‐spline basis of dimension k = 15, larger than required, with the smoothing parameter estimated by restricted maximum likelihood.
**Table S7:** MWF Summaries as scalar covariates.
This table presents a sample of the possible combinations of models evaluated. Models were fit to assess whether any of these MWF covariates contributed meaningfully to the model. VIF is the variable inflation factor. * denotes a *p*‐value. Models including only one of these variables were also evaluated but did not provide adequate results. The other scalar covariates were held fixed across models to allow for consistent comparison of model performance.
**Table S8:** Coefficients for the model with all scalar covariates (full model) and the model after AIC‐based backward stepwise selection (reduced model). Both contain the complete 10‐function B‐spline basis for the functional MWF covariate, which was not eligible for removal. Covariates removed by selection are shown as dashes (−). *p*‐values for covariates retained by selection do not account for the selection step and are consequently anti‐conservative (Freedman 1983; Harrell 2015). Individual basis coefficients are reported for completeness and are not interpreted individually; inference on the functional covariate is via the joint test. PRL, paramagnetic rim lesion; Treatment (low/high vs. none).
**Table S9:** Variance inflation factors, final model.
Variance inflation factors for the final model. Values for the scalar covariates were uniformly low (all ≤ 1.58), indicating negligible collinearity among the interpreted covariates. Values for the B‐spline basis functions were higher (3.40–10.12), as expected given the overlapping support of adjacent basis functions and the monotone form of the percentile functions; because individual basis coefficients are not interpreted, this does not affect inference on the functional covariate. PRL, paramagnetic rim lesion; VIF, variance inflation factor.
**Figure S1:** Regression diagnostic plots for the final model. (Top left) Residuals against fitted values, with a horizontal reference line at zero; the scatter shows no systematic trend or change in spread. (Top right) Normal quantile–quantile plot of the standardized residuals, with the line of identity in red; points lie close to the line apart from minor departures at the extreme tails. (Bottom left) Scale–location plot of the square root of the absolute standardized residuals against fitted values, with a smoothing curve; residual spread is approximately constant, increasing only slightly at the highest fitted values. (Bottom right) Standardized residuals against leverage, with a smoothing curve; observations of higher leverage have correspondingly small residuals, and no observation approaches an influential Cook's distance. Diagnostics are shown for the final model comprising the selected scalar covariates and the 10‐function B‐spline representation of the functional MWF covariate.

**Table S10:** Description of Cohort at Baseline and Year 4.
BVMT‐*R*, brief visuospatial memory test—revised; CVLT‐II, California verbal learning test—second edition; EDSS, expanded disability status scale; SDMT, symbol digit modalities test.

## Data Availability

Data will be shared subject to a formal agreement and any necessary institutional approvals, ensuring compliance with data use policies and confidentiality requirements.
