# Supplementary material for: Four‐Year Cognitive Outcomes in Multiple Sclerosis: Associations With Lesion Myelin Integrity
Source: Ann Clin Transl Neurol. 2026 Nov 4:10.1002/acn3.70577. Online ahead of print. doi: 10.1002/acn3.70577 (PMC13736402; doi:10.1002/acn3.70577)
Supplement: Supplementary file 1 [file ACN3-9999-0-s001.docx]

| Clinical Characteristics of Cohort | Baseline | Year 4 |
| --- | --- | --- |
| **EDSS,** Median [IQR] | 1.0 [0.0, 2.0] | 1.0 [0.0, 2.0] |
| Min – Max | 0.0 – 6.5 | 0.0 – 6.5 |
| **BVMT-R Total Score,** Mean (SD) | 24.16 (6.03) | 24.76 (6.92) |
| Minimum – Maximum | 9 - 36 | 7 - 36 |
| **CVLT-II Total Score,** Mean (SD) | 52.72 (8.50) | 57.21 (11.64) |
| Minimum – Maximum | 31 - 72 | 30 - 78 |
| **SDMT Total Score,** Mean (SD) | 56.11 (11.52) | 58.75 (12.81) |
| Minimum – Maximum | 29 – 93 | 11 - 96 |

**Table S10 Description of Cohort at Baseline and Year 4**

*Abbreviations:* EDSS = Expanded Disability Status Scale; BVMT-R = Brief Visuospatial Memory Test – Revised; CVLT-II = California Verbal Learning Test – Second Edition; SDMT = Symbol Digit Modalities Test.
