# Supplementary material for: Four‐Year Cognitive Outcomes in Multiple Sclerosis: Associations With Lesion Myelin Integrity
Source: Ann Clin Transl Neurol. 2026 Nov 4:10.1002/acn3.70577. Online ahead of print. doi: 10.1002/acn3.70577 (PMC13736402; doi:10.1002/acn3.70577)
Supplement: Supplementary file 2 [file ACN3-9999-0-s002.docx]

**Supplementary Material**

**Title: Four-Year Cognitive Outcomes in Multiple Sclerosis: The Role of Chronic Lesion Pathology and Structural MRI Measures**

**Corresponding Author:** Susan Gauthier, DO, MPH. **Email**: [sag2015@med.cornell.edu](mailto:sag2015@med.cornell.edu)

1. **The Modelling approach**

The goal of this study was to investigate the associations between clinical variables (scalar covariates), conventional global structural MRI metrics (scalar covariates), lesion-based myelin water fraction measures (functional covariate), and cognitive performance at four years post-assessment (response variable). In this section, we provide detailed specifications of the functional regression model, highlighting the role of the basis function in capturing complex relationships between lesion-based features and a cognitive outcome.

Each patient’s myelin water fraction varies from one lesion to another. Summarizing this variation with a single value such as the mean or median discards information about the shape of the distribution, and the choice of summary assumes which part of the distribution is relevant to the outcome. Functional regression addresses this by treating each patient’s entire lesion MWF distribution as a function, rather than reducing it to a few numbers. The regression then estimates how the association with the outcome varies across the distribution, allowing the data to indicate which features of it carry the association.

**1.1 Scalar and functional covariates**

Our **primary endpoint** was the Symbol Digit Modalities Test (SDMT) score at 4 years, modeled as a function of both **scalar and functional** covariates. **Scalar covariates** included baseline SDMT, age at baseline, disease duration at baseline, sex, and treatment at baseline. Baseline and follow-up scalar MRI covariates were also considered, including change in cortical thickness (Δct = cortical thickness at year 4 minus cortical thickness at baseline), change in normalized thalamic volume (Δntv= normalized thalamic volume at year 4 minus normalized thalamic volume at baseline), the presence of paramagnetic rim lesions (PRLs) at baseline (Y/N), and baseline lesion burden measured by both the number of FLAIR hyperintense lesions and total FLAIR lesion volume.

To maintain a parsimonious model and avoid potential collinearity between highly correlated baseline and follow-up measures, we used change in cortical thickness (Δct) and change in normalized thalamic volume (Δntv) as covariates rather than including both baseline and follow-up measures. This approach also reduced the number of parameters while providing a direct measure of association with structural change.

**The functional covariate** was myelin water fraction (MWF) at baseline, defined over lesion and summarized using its empirical lesion-percentile function. Specifically, for each patient, the MWF values of all lesions were summarized as an empirical percentile function. Let ${MWF}_{i}(q)$ denote the $q^{th}$ percentile of the lesion MWF distribution for patient $i$, for$q in [0, 1$]. Thus ${MWF}_{i}(0.5)$ is the patient's median lesion MWF, ${MWF}_{i}(0.70)$ is the value exceeded by only the most myelinated $30\%$of their lesions, and so on.

The percentile representation has two properties that make it suitable here. It places every patient on a common domain regardless of how many lesions they have, and it preserves the ordering of values within the distribution. Percentile functions are monotone non-decreasing by construction. Each function was evaluated on a common grid of $G$ equally spaced points. Because the resulting percentile functions are piecewise linear between observed values, evaluation on a fine grid improves the accuracy of numerical integration without adding information beyond that contained in each patient's lesions.

**1.2 The functional regression model**

Let $q\in\left[ 0,1 \right]$ index the percentiles of each patient MWF distribution, $MWF(q)$. **The functional regression model** takes the form:

$SDMT_{\left\{ Year 4 \right\}}= \boldsymbol{X\beta}+ \int_{0}^{1} MWF\left( q \right)\cdot\beta\left( q \right)dq +\epsilon$	(Eq. 1)

where $\boldsymbol{X}$ is the design matrix of scalar covariates (baseline SDMT, age at baseline, disease duration, sex, treatment, Δct, Δntv, the presence of PRLs, the number of FLAIR hyperintense lesions, and total FLAIR lesion volume), $\boldsymbol{\beta}$ denotes the corresponding scalar coefficients. $\beta\left( q \right)$ is the coefficient function for $MWF (q)$, and $\epsilon$ is the residual error term.

This model captures both the fixed effects of scalar covariates and the continuous variation across the MWF quantile curve. $\beta(q)$describes the association between SDMT and lesion $MWF$ at percentile$q$, while the rest of the distribution is fixed. The integral combines these contributions across the whole distribution, so the model uses every percentile rather than a selected subset. β(q) is approximated by a basis function (see S1.3).

This functional regression framework [1-2] was also applied to evaluate secondary cognitive outcomes, including California Verbal Learning Test-Second Edition (CVLT-II) and Brief Visuospatial Memory Test-Revised (BVMT-R) scores at follow-up.

**1.3. The Role of Basis function**

The coefficient function is represented as a weighted sum of $K$ known basis functions (Eq. 2). $K$determines the flexibility of the estimated coefficient function: A small $K$ constrains $\beta\left( q \right)$to simple shapes, while a larger$K$ permits more complex ones.

$\beta(q) = \sum_{\boldsymbol{k=1}}^{\boldsymbol{K}} \boldsymbol{\theta}_{\boldsymbol{k}} \boldsymbol{B}_{\boldsymbol{k}}\boldsymbol{(q)}$	(Eq. 2)

Eq. 2 in Eq. 1 turns the model into a linear model (see Eq. 3), where each$B_{k}(q)$ is a basis function defined on [0, 1], and $\theta_{k}$ is its corresponding coefficient. This representation allows flexible modeling of $\beta(q)$ while controlling smoothness [1].

$SDMT_{\left\{ Year 4 \right\}}= \boldsymbol{X\beta}+\sum_{k=1}^{K} \theta_{k} \left[ \int_{0}^{1} MWF\left( q \right)\cdot B_{k}\left( q \right)dq \right] +\epsilon$	(Eq. 3)

Eq. 3 is therefore an ordinary linear model in the scalar covariates. The bracketed integral, evaluated for each patient, forms one column of the design matrix for the functional block, and the $\theta_{k}$ are estimated by least squares. The coefficient function is then reconstructed from the estimated $\theta_{k}$ (Eq. 2).

Basis coefficients$\theta_{k}$ collectively determine the shape of $\beta(q)$. $\theta_{k}$ jointly describe a single curve and are not separately interpretable. Since percentile functions are monotone, the scores, $\int_{0}^{1} MWF\left( q \right)\cdot B_{k}\left( q \right)dq$, are strongly correlated across $k$. This is intrinsic to percentile-function covariates, and it means individual$\theta_{k}$ are weakly identified even when the joint effect is well estimated.

**Choice of basis families:** Many basis systems could in principle be used. We considered two, B-splines [3] and Bernstein polynomials [4], which are well suited to this setting on theoretical grounds. Both are defined on a closed bounded interval and can be placed directly on the percentile domain [0,1] without extrapolation beyond the range of the data. Both are non-negative and form a partition of unity, and both possess variation-diminishing properties, so that the reconstructed $\beta\left( q \right)$does not oscillate more than its coefficient sequence, a relevant property given that percentile functions are monotone and strongly autocorrelated across $q$. Bernstein polynomials are in addition the constructive basis of the Weierstrass approximation theorem, and therefore approximate any continuous function on [0,1] uniformly as $K$ increases. The two families differ in exactly the global support property. Bernstein basis functions have global support, so each coefficient influences the estimate across the whole domain and the resulting $\beta(q)$ is globally smooth. B-spline basis functions have local support, so each coefficient influences only a limited span of percentiles and localized features can be represented. Comparing the two therefore bears directly on whether any association is a global property of the distribution or is confined to part of it.

**1.4. Model Advantages**

This approach allows us to move beyond global averages and capture the distributional heterogeneity of lesion pathology, providing a more nuanced view of how specific lesion characteristics relate to cognitive outcomes. By integrating both scalar and functional features, the model can detect complex, patient-specific relationships that might be missed by standard regression methods.

Overall, this method enhances both the precision and the interpretability of lesion-level data to cognitive outcomes, highlighting subtle, clinically meaningful patterns that may inform prognosis and targeted interventions. This approach is advantageous compared with using a global MWF measure, as it preserves all the lesion maps and enables the identification of localized, complex relationships, potentially offering deeper biological insights.

**Prior applications of functional and distributional regression to MRI.** The representation of a within-region intensity distribution as a functional object has been applied to magnetic resonance imaging, most directly in radiomics. [5] developed quantile functional regression and applied it to an MRI-based radiomic study of glioblastoma, in which the distribution of voxel intensities within each tumor was represented as a quantile function and related to clinical and genetic covariates; their motivation, that extracting histogram summary features imposes a multiple-comparison burden and discards information contained in the shape of the distribution, parallels the rationale for the present analysis. The direction of the regression differs: the quantile function is the response in their formulation and the covariate in ours. Within multiple sclerosis, the treatment of myelin-sensitive intensity distributions as multivariate objects rather than as summary parameters dates to the analysis of magnetization transfer ratio histograms by principal components [6]. Related scalar-on-quantile-function approaches, using subject-specific quantile functions as covariates, have been applied outside imaging, for example to digital gait biomarkers in Alzheimer's disease [7]. To our knowledge, functional regression has not previously been applied to the distribution of myelin water fraction, nor used to relate it to longitudinal cognitive outcomes.

1.5. **Modeling Strategy and Estimation Steps**

The estimation procedure involves several sequential steps. Table S1 lists the order of operations explicitly, including definition of covariates values, estimation, variable selection, testing the functional effect and assessment of robustness.

| **Step** | **Procedure** | **Implementation** | | **Output** |
| --- | --- | --- | --- | --- |
| 1 | Extract MRI covariates | MRI Scanning Protocol  QSM and MWF Map Extraction  Image Co-Registration  Brain Tissue Segmentation  Lesion Segmentation and Paramagnetic Rim Lesion Identification | | For each patient we collected: MWF values (one per lesion), Δct, Δntv, PRLs, n.FLAIR, and total vol. FLAIR |
| 2 | Construct each patient's empirical percentile function on a common grid | Empirical percentile functions were computed as type 7 quantiles (Hyndman and Fan, 1996), evaluated at G = 101 equally spaced points on [0, 1]. Type 7 quantiles interpolate linearly between adjacent order statistics, so that the values at q = 0 and q = 1 are the patient's lowest and highest lesion MWF values, respectively. | | n x G matrix of percentile functions where n is the total number of patients and G is the grid size. |
| 3 | Specify the basis for $\beta(q)$ | B-spline and Bernstein polynomial bases, K = 4 to 15, fda package. Each candidate containing all scalar covariates and the complete basis block. | | Candidate fits |
| 4 | Select the basis specification. | **Numerical admissibility criteria**: κ, detection of aliased (rank-deficient) coefficients.  **Fit and parsimony criteria:** Adjusted R², AIC, BIC, generalized cross-validation.  **Sign changes, roughness** were introduced in revision and were not used in selection. | | Diagnostics reported in Table S2 |
| 5 | Compute the integrated basis scores | Trapezoidal quadrature over the grid | n × K matrix of scores (bracketed term in Eq. 3) | |
| 6 | Fit the model | Ordinary least squares | Estimates of $\beta$ and $\theta_{k}$ | |
| 7 | Select scalar covariates | Stepwise AIC-based selection (stepAIC, MASS), applied once; basis coefficients held fixed and never eligible for removal | Final set of scalar covariates | |
| 8 | Test the functional effect | Joint F-test with $\alpha$= 0.05  $H_{0}: \theta_{1}=\theta_{2}=\ldots=\theta_{K}=0$ , versus  $H_{a}:At least one \theta_{k}\neq0, k=1,\ldots,K$ | p-value | |
| 9 | Reconstruct the coef. function | Eq. 2 with estimated $\theta_{k}$ | Estimated$\beta(q)$ | |
| 10 | Visual Representation | Final model estimated (Eq. 3) | Figures 1-5 | |
| 11 | Assess robustness | Sensitivity analyses (Section 3) | See Table S3 | |

**Table S1. Estimation procedure**. Sequential steps in the construction of the functional covariate and estimation of the scalar-on-function regression model. Δct = cortical thickness at year 4 minus cortical thickness at baseline), Δntv= normalized thalamic volume at year 4 minus normalized thalamic volume at baseline, PRLs= the presence of paramagnetic rim lesions at baseline (Y/N), n.FLAIR= number of FLAIR hyperintense lesions and total vol. FLAIR=total FLAIR lesion volume. MWF, myelin water fraction; IQR, interquartile range; G, number of percentile grid points; K, number of basis functions; κ, condition number of the design matrix; $\theta_{k}$, basis coefficients; $\beta(q),$ coefficient function. Candidate specifications (step 4) each contained the full set of scalar covariates together with the complete basis block; no term was removed at this stage. Backward AIC-based selection (step 7) was applied once and to scalar covariates only, with the basis coefficients held fixed and at no stage eligible for removal. Full results for candidate specifications are given in Table S2, and sensitivity analyses in Table S3.

**1.6 Software**

All analyses were performed in R [8]. A significance level of 0.05 was used for the scalar and the joint test for functional covariates. Percentile functions and basis construction used the fda package [9]; stepwise selection used MASS [10]; penalized regression used refund [11] and mgcv [12]; and figures were produced with ggplot2 [13] and ggeffects [14].

2. **Selection of the basis specification.**

**2.1 Selection criteria**

Because the coefficient function is represented as a linear combination of $K$ basis functions and no roughness penalty was applied (Section S1.3), the basis family and dimension govern how much the estimated $\beta(q)$ is permitted to vary across the percentile domain. Candidate specifications were therefore compared before the final model was fitted. Candidates comprised two basis families, B-spline and Bernstein polynomial, at each dimension $K= 4 \mathrm{to} 15$. Each candidate contained the full set of scalar covariates together with the complete basis block; no term was removed at this stage, so that differences between candidates reflect basis family and dimension alone.

Specifications were assessed on two criteria: Numerical admissibility and Fit and parsimony criteria. Numerical admissibility criteria include the condition number of the design matrix, and detection of aliased (rank-deficient) coefficients. These depend only on the design matrix and not on the outcome. A specification exhibiting aliased coefficients is not estimable and will be excluded regardless of fit. The fit and parsimony criteria included adjusted R², the Akaike information criterion (AIC), the Bayesian information criterion (BIC), and the generalized cross-validation score. None of these criteria depends on the significance of the functional effect.

Two further quantities are reported in Table S2 to characterize the estimated coefficient function: The number of times the reconstructed$\beta(q)$crosses zero, and its roughness, defined as the integrated squared second difference. Both are reported for interpretation and were not used in selection.

**2.2 Results**

| **Basis** | **K** | **kappa** | **aliased** | **Adj. R²** | **AIC** | **BIC** | **GCV** | **Sign Changes** | **roughness** |
| --- | --- | --- | --- | --- | --- | --- | --- | --- | --- |
| bspline | 4 | 40.54 | FALSE | 0.657 | 604.30 | 646.42 | 57.06 | 2 | 0.03 |
| bspline | 5 | 62.38 | FALSE | 0.657 | 605.14 | 649.74 | 57.92 | 2 | 0.04 |
| bspline | 6 | 68.29 | FALSE | 0.654 | 606.76 | 653.83 | 59.32 | 4 | 2.93 |
| bspline | 7 | 92.70 | FALSE | 0.649 | 608.92 | 658.47 | 61.17 | 2 | 1.55 |
| bspline | 8 | 100.71 | FALSE | 0.658 | 607.06 | 659.08 | 60.27 | 7 | 247.80 |
| bspline | 9 | 109.38 | FALSE | 0.653 | 609.29 | 663.79 | 62.25 | 5 | 401.46 |
| bspline | 10 | 157.38 | FALSE | 0.673 | 604.25 | 661.22 | 59.22 | 6 | 3301.10 |
| bspline | 11 | 168.49 | FALSE | 0.671 | 604.29 | 663.73 | 59.62 | 8 | 8800.84 |
| bspline | 12 | 167.25 | FALSE | 0.670 | 604.28 | 666.20 | 60.75 | 8 | 12469.84 |
| bspline | 13 | 252.78 | FALSE | 0.669 | 605.25 | 669.64 | 59.35 | 8 | 12049.12 |
| bspline | 14 | 262.31 | FALSE | 0.668 | 605.16 | 672.03 | 59.83 | 8 | 9912.43 |
| bspline | 15 | 291.30 | FALSE | 0.668 | 605.19 | 674.54 | 62.90 | 8 | 9350.85 |
| bernstein | 4 | 40.54 | FALSE | 0.657 | 604.30 | 646.42 | 57.06 | 2 | 0.03 |
| bernstein | 5 | 129.24 | FALSE | 0.658 | 605.05 | 649.64 | 57.58 | 2 | 0.04 |
| bernstein | 6 | 285.98 | FALSE | 0.653 | 606.97 | 654.04 | 59.46 | 2 | 3.21 |
| bernstein | 7 | 770.83 | FALSE | 0.649 | 608.92 | 658.47 | 61.17 | 4 | 9.21 |
| bernstein | 8 | 1584.13 | FALSE | 0.652 | 608.83 | 660.86 | 61.50 | 7 | 549.92 |
| bernstein | 9 | 3621.82 | FALSE | 0.650 | 610.12 | 664.62 | 62.84 | 6 | 1970.52 |
| bernstein | 10 | 8523.94 | FALSE | 0.664 | 606.83 | 663.80 | 60.98 | 9 | 15819.46 |
| bernstein | 11 | 19837.89 | FALSE | 0.663 | 607.63 | 667.08 | 62.02 | 8 | 38675.90 |
| bernstein | 12 | 44868.28 | FALSE | 0.666 | 607.52 | 669.45 | 62.46 | 10 | 25136.94 |
| bernstein | 13 | 96708.66 | FALSE | 0.676 | 605.17 | 669.58 | 61.35 | 8 | 6282.02 |
| bernstein | 14 | 223882.29 | FALSE | 0.676 | 605.86 | 672.75 | 62.41 | 8 | 6063.67 |
| bernstein | 15 | 587755.69 | FALSE | 0.684 | 603.77 | 673.13 | 61.54 | 8 | 6072.86 |

**Table S2. Candidate specifications for the coefficient function**. κ, condition number of the design matrix; Aliased, presence of rank-deficient (aliased) coefficients. Adj. R², adjusted R²; AIC, Akaike information criterion; BIC, Bayesian information criterion; GCV. Sign changes count crossings of zero by the reconstructed β(q), and roughness is its integrated squared second difference; both were introduced in revision for interpretation and were not used in selection.

Table S2 shows that no candidate specification exhibited aliased coefficients. Condition numbers ($\kappa$) increased approximately monotonically with $K$ for both basis, indicating progressively weaker identification of individual basis coefficients at higher dimensions, although all specifications remained estimable. Bernstein polynomial specifications exhibited substantially higher condition numbers than B-spline specifications at comparable dimension (κ =38675.90 at K = 11). The B-spline family was therefore preferred on numerical grounds, independent of model fit.

Adjusted R² varies little across specifications, while the roughness of the reconstructed coefficient function increases by several orders of magnitude with K. Additional basis dimensions therefore yield negligible gain in fit while producing increasingly oscillatory coefficient functions, consistent with the collinearity described in Section 1.3. basis specifications.

The two best specifications were B-spline k=4 and k=10 and they are indistinguishable on predictive criteria (ΔAIC = 0.05; GCV 57.06 versus 59.22). K = 10 required six additional parameters, which BIC penalized (ΔBIC = 14.8). We report (B-spline, K = 10) as the primary analysis. Sections 3 runs a comprehensive sensitivity analysis.

3. **Sensitivity Analysis**

The estimation procedure described in Section 1 involves choices that are not determined by the data alone: The family and dimension of the basis representing $\beta(q)$, and the set of scalar covariates retained. Each could in principle influence the conclusions. We therefore checked the robustness of our findings regarding each of these choices.

Table S3 lists the full set of analyses and subsections report the results. Sections 3.1 to 3.3 vary the modelling framework of Section 1 by estimation procedure and covariate representation. Sections 3.4 and 3.5 characterize the data and verify assumptions.

|  | **Procedure** | **Implementation** |
| --- | --- | --- |
| 3.1 | Functional principal components | Assess dependence on the basis by using an orthogonal, data-derived representation |
| 3.2 | Penalized estimation | Assess whether the association depends on the absence of a roughness penalty |
| 3.3 | Model with MWF covariates as a scalar summary representation | Assess whether a set of scalar summaries of the distribution conveys equivalent information |
| 3.4 | Sensitivity to variable selection | Compare the selected model with the full model to check sensitivity of the associations to variable selection. |
| 3.5 | Model assumptions | Verify distributional assumptions and assess sensitivity to extreme residuals (if any) |

**Table S3. Sensitivity analyses procedure.** The table presents several sensitivity steps relative to the primary model. The primary model is the scalar-on-function regression described in Section 1: SDMT at four years regressed on the lesion myelin water fraction percentile function, represented by a B-spline basis, together with baseline cognitive score, age, disease duration, sex, treatment, change in cortical thickness, change in normalized thalamic volume, presence of at least one paramagnetic rim lesion, number of FLAIR lesions, and total FLAIR lesion volume. In every section the functional covariate was retained by design and was at no stage eligible for removal. β(q), coefficient function; REML, restricted maximum likelihood; MWF, myelin water fraction.

**3.1	Functional principal component analysis**

Using individual percentiles as separate covariates would place near-redundant covariates in the same model, producing unstable coefficients. Representing the MWF distribution as a function rather than as a set of values is an improvement as the basis expansion summarizes many correlated ordinates in K coefficients, borrowing strength across neighboring percentiles instead of treating them as independent quantities. The correlation is reduced by this representation but not removed. Integrated scores computed against any fixed basis remain strongly correlated with one another, because the underlying function is monotone; this is intrinsic to the data, not to the basis chosen (Section 1.3).

We therefore repeated the analysis using functional principal components [15] of the lesion MWF percentile functions. Because the components are derived from the data by eigenvalue/ eigenvector decomposition of the covariance operator, they are mutually orthogonal by construction. The correlation among covariates is thereby removed rather than merely reduced, the design is perfectly conditioned, and no basis dimension must be selected. The decomposition was applied to the centered percentile functions, on the MWF scale. First to four components are reported.

The first two components accounted for 77.7% and 16.5% of the variance in the lesion MWF percentile functions (94.2% cumulative; Table S4). The first component, which reflects the overall level of the distribution, showed no association with four-year SDMT in any model (β = 0.03 to 0.04, p = 0.83 to 0.87). The second was significantly associated (β = 0.43, 95% CI 0.14 to 0.72), and the estimate was essentially unchanged by the inclusion of third and fourth components (Table S5). The joint test of the first two components was significant (p = 0.008). The second component is a monotone contrast weighing the lower half of the distribution against the upper half, crossing zero at approximately the median. Higher scores were associated with better SDMT. The association is therefore with a range of lesion myelin content rather than with its overall mean as reflected by the correlation between the FPC2 and the IQR, and FPC2 and Mid-range of the scores (-0.803 and -0.75, respectively) . We report this analysis as a check on dependence of our findings on the basis specification rather than as an independent replication.

| **FPC** | **Variance explained (%)** | **Cumulative (%)** |
| --- | --- | --- |
| FPC1 | 77.73 | 77.73 |
| FPC2 | 16.47 | 94.20 |
| FPC3 | 2.21 | 96.41 |
| FPC4 | 1.49 | 97.90 |
| FPC5 | 0.65 | 98.55 |

**Table S4. Variance explained by functional principal components**

Proportion of variance in the lesion myelin water fraction percentile functions accounted for by each functional principal component. Components were obtained by eigenvalue decomposition of the covariance operator of the centered percentile functions across the patients in the analytic sample. FPC is the functional principal component.

|  | **1 Component Model** | **2 Components Model** | **3 Components Model** | **4 Components Model** |
| --- | --- | --- | --- | --- |
| FPC1 | 0.01 (-0.13, 0.15) | 0.01 (-0.12, 0.15) | 0.01 (-0.12, 0.15) | 0.01 (-0.13, 0.15) |
| FPC2 | — | 0.43 (0.14, 0.72) | 0.43 (0.14, 0.72) | 0.43 (0.14, 0.72) |
| FPC3 | — | — | -0.46 (-1.24, 0.33) | -0.45 (-1.24, 0.33) |
| FPC4 | — | — | — | 0.20 (-0.78, 1.19) |
| Joint test p | 0.457 | 0.008 | 0.012 | 0.024 |
| Adj. R² | 0.500 | 0.536 | 0.537 | 0.533 |

**Table S5. Association between functional principal component scores and four-year SDMT.** Coefficients for functional principal component scores in models of Symbol Digit Modalities Test score at four years, with 95% confidence intervals in parentheses. Each model additionally contains the full set of scalar covariates listed in section 1. Models with one to four components are reported so that the stability of each estimate across specifications is apparent; no single number of components was selected. Dash (—) indicates the component was not included in that model. The joint test compares each model with the corresponding model omitting FPC scores. Because component scores are scaled by the eigenvalue decomposition of the covariance operator, coefficient magnitudes are not directly comparable between components. FPC is functional principal component; Adj. R² is adjusted R².

**3.2	Penalized estimation**

As a further check we fitted a roughness-penalized scalar-on-function regression [16], representing β(q) with a B spline basis of dimension k = 15 and estimating the smoothing parameter by REML [17]. In this formulation the number of basis functions does not have to be selected, the roughness penalty rather than $K$ governs the flexibility of β(q), and results are insensitive to $K$ provided it is sufficiently large (effective df 2.0–2.6 across k = 4 to 25).

| **Quantity** | **Value** |
| --- | --- |
| Basis | B-spline |
| Basis dimension supplied (k) | 15 |
| Smoothing parameter selection | REML |
| Effective degrees of freedom, beta(q) | 2.57 |
| Reference df | 2.91 |
| Test statistic (F) | 2.67 |
| p-value, joint test of beta(q)=0 | 0.039 |
| Smoothing parameter (lambda) | 208.6 |

**Table S6.** **Penalized scalar-on-function regression.** Roughness-penalized scalar-on-function regression of four-year SDMT on the lesion MWF percentile function, adjusting for the full scalar covariate set. The coefficient function was represented with a B-spline basis of dimension k = 15, larger than required, with the smoothing parameter estimated by restricted maximum likelihood.

The penalty selected a smooth coefficient function of modest complexity (effective df = 2.57, well below the supplied dimension of 15), and the functional effect was significant (F = 2.67, p = 0.039).

Three approaches, an orthogonal decomposition, a low-dimensional basis, and a data-driven roughness penalty, agree that the association between the lesion MWF distribution and four-year cognitive performance is real and confined to a small number of dimensions. Notably, the penalty regularizes precisely the flexibility that produced the oscillatory unpenalized estimate at K = 10, and the association survives, indicating that it is not an artefact of overfitting.

**3.3	Model with MWF covariates as a scalar summary representation**

We evaluated alternative model specifications incorporating different summaries of the MWF distribution (mean, SD, and quartiles). Each column represents an independent model; the models were not intended for direct comparison, but rather to assess whether simple regression approaches provided evidence of an association with the outcome.

Table S7 shows that none of the MWF measures demonstrated a statistically significant association across the alternative specifications. In addition, several MWF measures exhibited substantial multicollinearity (see models 2-5). Models including only one of these MWF summary measures were also evaluated and did not provide evidence of an association. These sensitivity analyses therefore did not support the use of mean, SD, or individual quartile measures as alternative model specifications. The remaining scalar covariates were kept fixed across the analyses to facilitate consistent evaluation of these alternative approaches.

|  | **Model 1** | | | **Model 2** | | | **Model 3** | | | **Model 4** | | | **Model 5** | | |
| --- | --- | --- | --- | --- | --- | --- | --- | --- | --- | --- | --- | --- | --- | --- | --- |
|  | $\boldsymbol{\beta}$ | $\boldsymbol{VIF}$ | | $\boldsymbol{\beta}$ | $\boldsymbol{VIF}$ | | $\boldsymbol{\beta}$ | $\boldsymbol{VIF}$ | | $\boldsymbol{\beta}$ | $\boldsymbol{VIF}$ | | $\boldsymbol{\beta}$ | $\boldsymbol{VIF}$ | |
| MWF mean | -0.180 (0.793) | | 1.03 | - | | - | - | | - | 5.156 (0.212) | | 36.67 | 3.10 (0.599) | | 74.49 |
| MWF sd | -2.145 (0.057) | | 1.02 | - | | - | -1.587 (0.623) | | 6.01 | -2.50 (0.354) | | 3.51 | -1.64 (0.613) | | 6.02 |
| MWF Q1 | - | | - | 0.961 (0.514) | | 10.77 | 0.243 (0.906) | | 11.41 | 2.01 (0.482) | | 21.77 | 1.45 (0.639) | | 25.26 |
| MWF Q2 | - | | - | 2.54 (0.288) | | 13.53 | 2.40 (0.32) | | 13.73 | 2.98 (0.273) | | 17.35 | 3.06 (0.264) | | 17.40 |
| MWF Q3 | - | | - | -3.55 (0.053) | | 15.34 | -2.73 (0.218) | | 12.29 | - | - | | -1.54 (0.625) | | 24.97 |
| Adj. R² | 0.543 | | | 0.554 | | | 0.55 | | | 0.55 | | | 0.528 | | |

**Table S7. MWF Summaries as scalar covariates.** This table presents a sample of the possible combinations of models evaluated. Models were fit to assess whether any of these MWF covariates contributed meaningfully to the model. VIF is the variable inflation factor. () denotes a p-value. Models including only one of these variables were also evaluated but did not provide adequate results. The other scalar covariates were held fixed across models to allow for consistent comparison of model performance.

**3.4	Sensitivity to variable selection**

To assess whether the reported associations depended on the stepwise selection of scalar covariates, Table S8 presents the full and reduced models, with the functional MWF block forced in and not eligible for removal in either specification. Estimates were stable across the two models (presence of a paramagnetic rim lesion, −4.77 versus –4.397; baseline SDMT, 0.644 versus 0.673; age, −0.278 versus −0.294, change in cortical thickness 45.410 to 43.882).

| **Term** | **Full model** | | **Final Model** | |
| --- | --- | --- | --- | --- |
|  | **Estimate** | **p** | **Estimate** | **p** |
| ***Scalar covariates*** |  |  |  |  |
| (Intercept) | 42.066 | <0.001 | 43.114 | <0.001 |
| Baseline SDMT | 0.644 | <0.001 | 0.673 | <0.001 |
| Age | -0.278 | 0.011 | -0.294 | <0.001 |
| Disease duration | -0.228 | 0.124 | — | — |
| Treatment (low) | -0.886 | 0.790 | — | — |
| Treatment (high) | -2.115 | 0.516 | — | — |
| PRL present | -4.774 | 0.023 | -4.397 | 0.019 |
| Delta cortical thickness | 45.410 | 0.015 | 43.882 | 0.014 |
| Delta normalized thalamic volume | 2746.160 | 0.164 | — | — |
| Lesion number | 0.010 | 0.865 | — | — |
| Lesion Vol (log) | -0.958 | 0.408 | — | — |
| ***Functional MWF term*** |  |  |  |  |
| MWF basis 1 | 1.558 | 0.495 | 1.301 | 0.115 |
| MWF basis 2 | -1.020 | 0.351 | -1.136 | 0.226 |
| MWF basis 3 | -1.305 | 0.685 | -1.219 | 0.232 |
| MWF basis 4 | 2.324 | 0.270 | 2.267 | 0.116 |
| MWF basis 5 | -2.353 | 0.710 | -2.648 | 0.107 |
| MWF basis 6 | 4.860 | 0.005 | 4.718 | 0.006 |
| MWF basis 7 | -2.347 | 0.106 | -1.830 | 0.198 |
| MWF basis 8 | -1.417 | 0.153 | -1.106 | 0.216 |
| MWF basis 9 | -0.557 | 0.829 | -0.965 | 0.372 |
| MWF basis 10 | 0.392 | 0.863 | 0.320 | 0.671 |
| Joint test of functional terms (p) | 0.02 | | 0.04 | |
| Adjusted R-squared | 0.673 | | 0.719 | |
| AIC | 604.249 | | 594.841 | |

**Table S8.** Coefficients for the model with all scalar covariates (full model) and the model after AIC-based backward stepwise selection (reduced model). Both contain the complete 10-function B-spline basis for the functional MWF covariate, which was not eligible for removal. Covariates removed by selection are shown as dashes (-). p-values for covariates retained by selection do not account for the selection step and are consequently anti-conservative (Freedman 1983; Harrell 2015). Individual basis coefficients are reported for completeness and are not interpreted individually; inference on the functional covariate is via the joint test. PRL, paramagnetic rim lesion; Treatment (low/high versus none)

The functional covariate is assessed by a single joint test of all ten basis coefficients ($H₀: \beta(q) = 0 for all q)$, rather than by the ten coefficients individually, since the coefficients jointly describe one coefficient function and are not separately meaningful. This joint test was consistent across the two models (p = 0.02 full, p = 0.04 selected). The improvement in AIC after selection (604.25 to 594.84) reflects the removal of scalar covariates that did not contribute to fit; because the two models contain different scalar covariate sets, this difference concerns the scalar terms and not the functional covariate. The reported associations were therefore not artefacts of the variable-selection procedure.

**3.5	Model assumptions**

Diagnostic plots for the final model are shown in Figure S1. Residuals were approximately symmetrically distributed about zero across the range of fitted values, with no systematic trend, and the normal quantile–quantile plot showed residuals lying close to the theoretical line apart from minor departures at the extreme tails. The scale location plot indicated approximately constant residual variance, with only a slight increase at the highest fitted values. Leverage was modest throughout, although a small number of observations had leverage up to approximately 0.4, these had correspondingly small residuals, and no observation approached an influential Cook's distance. The single largest residual occurred at a point of low leverage and therefore exerted little influence on the fit. These diagnostics indicate that the model assumptions were adequately met and that no individual observation unduly influenced the results**.**

**Figure S1.** Regression diagnostic plots for the final model. (Top left) Residuals against fitted values, with a horizontal reference line at zero; the scatter shows no systematic trend or change in spread. (Top right) Normal quantile–quantile plot of the standardized residuals, with the line of identity in red; points lie close to the line apart from minor departures at the extreme tails. (Bottom left) Scale–location plot of the square root of the absolute standardized residuals against fitted values, with a smoothing curve; residual spread is approximately constant, increasing only slightly at the highest fitted values. (Bottom right) Standardized residuals against leverage, with a smoothing curve; observations of higher leverage have correspondingly small residuals, and no observation approaches an influential Cook's distance. Diagnostics are shown for the final model comprising the selected scalar covariates and the ten-function B-spline representation of the functional MWF covariate.

**Collinearity.** Variance inflation factors are reported in S9. Among the scalar covariates they were uniformly low (all ≤ 1.58: baseline SDMT 1.30, presence of a paramagnetic rim lesion 1.58, change in cortical thickness 1.20, change in normalized thalamic volume 1.25, age 1.17), indicating negligible collinearity among the interpreted covariates and stable estimation of their coefficients. The B spline basis functions showed higher factors (range 3.40 to 10.12), as expected given the overlapping support of adjacent basis functions and the monotone form of the percentile functions, which render neighboring integrated basis scores correlated. Because individual basis coefficients are not interpreted, this collinearity does not affect inference on the functional covariate, which rests on the joint test of all basis coefficients together and on the functional principal components and penalized analyses of Sections 1 to 3.

| **Term** | **VIF** |
| --- | --- |
| ***Scalar covariates*** |  |
| Baseline SDMT | 1.30 |
| PRL present | 1.58 |
| Delta cortical thickness | 1.20 |
| Delta normalized thalamic volume | 1.25 |
| Age | 1.17 |
| ***Functional MWF term*** |  |
| MWF basis 1 | 5.68 |
| MWF basis 2 | 6.62 |
| MWF basis 3 | 5.17 |
| MWF basis 4 | 8.19 |
| MWF basis 5 | 10.12 |
| MWF basis 6 | 9.75 |
| MWF basis 7 | 8.57 |
| MWF basis 8 | 3.81 |
| MWF basis 9 | 6.17 |
| MWF basis 10 | 3.40 |

**Table S9. Variance inflation factors, final model.** Variance inflation factors for the final model. Values for the scalar covariates were uniformly low (all ≤ 1.58), indicating negligible collinearity among the interpreted covariates. Values for the B-spline basis functions were higher (3.40–10.12), as expected given the overlapping support of adjacent basis functions and the monotone form of the percentile functions; because individual basis coefficients are not interpreted, this does not affect inference on the functional covariate. PRL, paramagnetic rim lesion; VIF, variance inflation factor.

**References**

1. Ramsay JOS, B.W. Functional Data Analysis, 2nd ed. New York, NY: Springer, 2005.
2. Morris JS. Functional Regression. Annu Rev Stat Appl 2015; 2:321–359.
3. de Boor C. A Practical Guide to Splines. Rev. ed. New York: Springer; 2001.
4. Farouki RT. The Bernstein polynomial basis: a centennial retrospective. Comput Aided Geom Des. 2012;29(6):379–419.
5. Yang H, Baladandayuthapani V, Rao AUK, Morris JS. Quantile function on scalar regression analysis for distributional data. J Am Stat Assoc. 2020;115(529):90–106.
6. Dehmeshki J, Ruto AC, Arridge S, Silver NC, Miller DH, Tofts PS. Analysis of MTR histograms in multiple sclerosis using principal components and multiple discriminant analysis. Magn Reson Med. 2001 Sep;46(3):600–9.
7. Ghosal R, Varma VR, Volfson D, et al. Distributional data analysis via quantile functions and its application to modeling digital biomarkers of gait in Alzheimer's disease. Biostatistics. 2023 Jul 14;24(3):539–61.
8. R Core Team (2025). _R: A Language and Environment for Statistical Computing. R Foundation for Statistical Computing, Vienna, Austria. <https://www.R-project.org/>
9. Ramsay J (2024). fda: Functional Data Analysis. R package version 6.1.8, <https://CRAN.R-project.org/package=fda>.
10. Venables WN, Ripley BD. Modern Applied Statistics with S. 4th ed. New York: Springer; 2002.
11. Goldsmith J, Scheipl F, Huang L, et al. refund: Regression with Functional Data. R package version [0.1-37]; [2024]. <https://CRAN.R-project.org/package=refund>
12. Wood SN. Generalized Additive Models: An Introduction with R. 2nd ed. Boca Raton, FL: Chapman and Hall/CRC; 2017.
13. Wickham H. ggplot2: Elegant Graphics for Data Analysis. 2nd ed. New York: Springer; 2016.
14. Lüdecke D. ggeffects: tidy data frames of marginal effects from regression models. J Open Source Softw. 2018;3(26):772.
15. Reiss PT, Ogden RT. Functional principal component regression and functional partial least squares. J Am Stat Assoc. 2007 Sep;102(479):984–96.
16. Goldsmith J, Bobb J, Crainiceanu CM, Caffo B, Reich D. Penalized functional regression. J Comput Graph Stat. 2011;20(4):830–51.
17. Wood SN. On p-values for smooth components of an extended generalized additive model. Biometrika. 2013 Mar;100(1):221–8.
